# Tobacco Use Among High School Students of West Bengal, India

**DOI:** 10.4103/0970-0218.42069

**Published:** 2008-07

**Authors:** Dechenla Tsering, Ranabir Pal, Aparajita Dasgupta

**Affiliations:** Department of Community Medicine, Sikkim Manipal Institute of Medical Sciences, 5^th^ Mile Tadong, Gangtok, Sikkim – 737 102, India. E-mail- dtsering16@gmail.com; 1 Department of Community Medicine, All India Institute of Hygiene and Public Health, Chittaranjan Avenue, Kolkata, West Bengal, India

Sir,

This study was conducted to estimate the prevalence, frequency and knowledge regarding the adverse effects of tobacco use among high school students of West Bengal, India.

From June 1^st^, 2003 to May 31^st^, 2004, we conducted a population-based cross-sectional study on students selected by a multistage random sampling. An anonymous self-administered pretested close-ended questionnaire was designed by adopting the questionnaire developed by the study team of the World health Organization (WHO, 1980)(1) with regard to both smoke and smokeless tobacco use. Of all urban and rural high schools in West Bengal, one school in each category was randomly selected. The study population comprised 478 students of class VIII, IX and X from two schools (urban school, 210 students; rural school, 268 students); of these students selected, 462 (urban school, 205 students; rural school, 257 students) were studied. The final response was obtained from 416 students (87.02%). After obtaining permission from school authorities and verbal informed consent from the subjects enrolled, the first author and the principal investigator collected the data from the students on the same day.

Overall prevalence was 9.61%; the prevalence among urban and rural students was 11.05% and 8.61%, respectively. Tobacco use among males (urban = 11.35% and rural = 15.04%) was higher than that among females (urban = 9.68% and rural = 0.90%). Tobacco use was more common among the rural male students although not significant (Z = 0.9, *P* > 0.05). The opposite was true among the female students (Z = 4.4, *P* < 0.05). Male students (13.14%) were associated more with tobacco use than the female students (2.82%). The current tobacco use ranged from 36.84% to 52.38% among the urban and rural students, respectively. The regular use of tobacco was higher among the rural students (14.29%) than the urban students (10.53%).

Current as well as regular use of tobacco was higher among urban as well as rural male students, whereas females were restricted to the first use [Table 1].

**Table 1:** Frequency of tobacco use among urban and rural students

Place	Number of ever users (%)			Number of current users (% of ever users)			Number of regular users (% of ever users)		
			
	Male	Female	Total	Male	Female	Total	Male	Female	Total
Urban (n = 19)	16 (11.35)	03 (9.68)	19 (100)	07 (43.75)	00	07 (36.84)*	02 (12.50)	00	02 (10.53)#
Rural (n = 21)	20 (15.04)	01 (0.90)	21 (100)	10 (50.00)	01 (100)	11 (52.38)*	03 (30.00)	00	3 (14.29)#

* X^2^ = 0.97; df = 1; *P* > 0.05

# X^2^ = 0.01; df = 1; *P* > 0.05

Note: Ever users: individuals using tobacco irrespective of the time and frequency. Current users: individuals using tobacco at least once during the past 30 d. Regular users: individuals using tobacco for 20 d or more during the past 30 d.

A majority of the urban and rural users were aware of the harmful effects of tobacco use. All the 19 urban users had the knowledge regarding the deleterious effects of tobacco use. A majority (76.19%) among the rural users also knew about such effects of tobacco use.

## Discussion

The prevalence rate of tobacco use in the present study shows results similar to those of other studies,(2,3) whereas in few studies, the prevalence rates are higher.(4) In this study, males were more likely than females to use all types of tobacco products; further, the studies conducted by other authors showed similar results.(5–8) The current and regular tobacco use (both smoke and smokeless) in any form among male students exceeded their female counterparts, which was evident from the results of similar studies.(4,5) Current as well as regular use was found more among the rural students, but opposite results were found in a study conducted in Kenya.(8) The variation in the results can be attributed to the study of different forms of tobacco in other studies.(9) A majority of the students used tobacco in spite of having the knowledge about their harmful effects; similar findings have been cited in other studies,(10) which is, unfortunately, very alarming.
